# Early negative affect in males and females with fragile X syndrome: implications for anxiety and autism

**DOI:** 10.1186/s11689-019-9284-y

**Published:** 2019-09-13

**Authors:** Carla A. Wall, Abigail L. Hogan, Elizabeth A. Will, Samuel McQuillin, Bridgette L. Kelleher, Jane E. Roberts

**Affiliations:** 1 Department of Psychology, University of South Carolina, 1512 Pendleton Street, Barnwell College, Suite #220, Columbia, SC 29208 USA; 2 Department of Psychological Sciences, Purdue University, 703 Third Street, West Lafayette, IN 47907-2081 USA

**Keywords:** Fragile X syndrome, Autism spectrum disorder, Anxiety, Negative affect, Sex differences

## Abstract

**Background:**

Fragile X syndrome (FXS) is a genetic disorder that is highly comorbid with anxiety and autism spectrum disorder (ASD). Elevated negative affect in young children has been associated with increased risk for both anxiety and ASD; however, these relations remain poorly understood in FXS.

**Methods:**

The present prospective longitudinal study examined the trajectory of negative affect from infancy through preschool in males and females with FXS and typical development and its relation to anxiety and ASD.

**Results:**

Results indicate a complex association reflecting group, developmental, and sex effects. Specifically, the group with FXS displayed a trajectory of increasing negative affect across age that was distinct from the typical controls. This atypical trajectory of negative affect in FXS was driven by sex effects in that males showed lower negative affect during infancy followed by steep increases across the toddler and preschool years whereas the females displayed a flatter trajectory. Finally, elevated negative affect predicted anxiety symptoms in males, but not females, with no relationship to ASD in males or females with FXS.

**Conclusions:**

The current work addresses the importance of studying the development of psychopathology in a specific neurogenetic population. Temperamental negative affect was shown to be an important early marker for anxiety in young children with FXS, with subtle differences observed between males and females.

## Background

Fragile X syndrome (FXS) is a rare genetic disorder caused by mutations on the *FMR1* gene and the ensuing under-expression of its associated fragile X mental retardation protein (FMRP; [1]). FXS is the most common inherited cause of intellectual disability, with an estimated prevalence of 1 in 4000–5000 males and 1 in 6000–8000 females [2]. Very few studies have included females in their samples or addressed questions about the clinical presentation of females with FXS, and the developmental profiles of very young females with FXS remain uncharacterized. The few studies that have been conducted show significant differences in clinical presentation between males and females with FXS because of the protective effects of the unaffected X chromosome in females [3].

Individuals with FXS, both males and females, are at heightened risk of comorbid diagnoses and disorders, especially anxiety and autism spectrum disorder (ASD). Current estimates indicate that 86% of males and 77% of females with FXS have an anxiety disorder [4–6], and 50–75% of males and 25% of females with FXS meet diagnostic criteria for ASD [4, 5, 7–10]. However, very little is understood about the earliest predictors of anxiety or ASD in FXS. There are known sex differences in prevalence and presentation in both ASD and anxiety; however, it is unknown whether these differences are exasperated or attenuated in FXS given the established sex differences in this population as well [11–13]. It remains unknown whether negative affect, a promising risk marker of anxiety and ASD in other populations, presents differently across very young males and females with FXS [14–16].

### Negative affect as a predictor of anxiety and ASD in non-FXS populations

Temperament refers to a characteristic “style” of behavior demonstrated to have strong heritable influence [17, 18]. Negative affect is a temperament domain characterized by negative emotions such as fear, sadness, or frustration, as well as difficulty being soothed [19]. Certain components of negative affect, such as fear and anger, become discernable at around 4–8 months of age [20]. Early negative affect, and fear in particular, has shown clinical utility as a predictor of later anxiety in neurotypical infants and toddlers. Further, negative affect can be measured reliably with parent report using measures such as the Rothbart Temperament Questionnaires, which are among the most widely-used measures of negative affect in infancy and childhood [15, 21, 22]. Because parent-report temperament measures are easily disseminable and administered, they are a useful tool for understanding trajectories early in life, and a large collection of research using the Rothbart scales has identified a relationship between high levels and increasing trajectories of negative affect predicting later anxiety [23–25]. Research has demonstrated that this relation holds throughout early life, with infant fear predicting toddlers’ anxiety levels [24], and toddlers’ negative affect predicting anxiety symptoms from 4 to 11 years of age [26]. Finally, neurotypical infants who show early social fear display later anxious behaviors [23, 25, 27]. Thus, negative affect is a powerful predictor of later clinical risks.

Previous work generally suggests that there are no biological sex differences in levels of early parent-reported negative affect in typically developing children [28]. However, some longitudinal work suggests that males and females exhibit differences in the relation between negative affect and other outcomes [24, 25, 29], and thus the clinical interpretation of negative affect may vary by sex. For example, female infants who later develop anxiety experience steeper increases in fear over time relative to males with anxiety [24]. In addition, steeper increases in infant fear have been associated with physiological markers of anxiety at 8 years in females but not males [25]. Other work has found sex differences in physiological markers of anxiety in females but not males with high internalizing symptoms; together, this work suggests that there may be multiple etiological pathways to anxiety symptoms that differ by sex [29]. Especially when taking biological sex into account, the predictive power of negative affect varies across different ages, pointing to the importance of studying temperament longitudinally. Importantly, it remains unknown whether sex differences in negative affect exist in populations with neurodevelopmental disorders.

Negative affect has been implicated as a useful predictor of later ASD diagnosis in a subset of children at elevated genetic risk for ASD, such as later-born siblings of children with ASD [30–32]. In longitudinal prospective studies of infant siblings of children with ASD, higher parent-reported negative affect and frustration in infancy has been repeatedly associated with ASD outcomes in toddlerhood [30, 32]. In addition, Garon and colleagues [31] used cluster analysis to determine that temperamental profiles including higher negative affect predicted ASD diagnosis in a high-risk cohort including males and females. Recent work has also demonstrated that clinic-referred toddlers with ASD tended to have temperamental profiles associated with higher negative affect as compared to their typical development (TD) peers; however, negative affect did not distinguish toddlers with ASD from those with general developmental delays, suggesting that higher negativity may not be a characteristic specific to ASD [33]. This work points to a number of temperamental profiles that are specific to certain phenotypes or disorders. However, unlike the anxiety literature, sex differences have been underexplored in prospective studies of temperament in children with later ASD.

### Negative affect as a predictor of anxiety and ASD in FXS

Preliminary work has begun to characterize atypical profiles of negative affect in males with FXS using behavioral and parent-report measures. For example, infants and toddlers with FXS exhibit atypical longitudinal patterns of facial and behavioral social fear, and these patterns are associated with withdrawal and ASD symptoms [34]. In contrast, increased social fear was found to predict elevated ASD symptoms in both preschool males with FXS and males with non-syndromic ASD despite different manifestations of social fear across groups [35]. Parent report measures of negative affect, in addition to being cost- and time-efficient measures of early development, have also shown utility from a risk-prediction standpoint. Initial cross-sectional work suggested that 3-year-old males with FXS have lower parent-reported levels of negative affect than their TD peers [36]. However, a prospective longitudinal study reported that infant and preschool-aged males with FXS exhibited increasing negative affect across age and that early negative affect predicted anxiety but not ASD symptoms [14]. Together, these studies suggest that parent-reported negative affect may have clinical utility for understanding anxiety risk in FXS, whereas behaviorally indexed social fear may be more closely related to ASD features.

Taken together, these findings provide proof of concept that early negative affect may provide useful clinical information in FXS, potentially aiding in identification of children most at risk for anxiety or ASD-related impairments. However, several gaps in the literature remain. First, no work to date has compared the trajectory of parent-reported negative affect in children with FXS to that of their TD peers, so it is unclear whether developmental changes in negative affect follow a normative pattern in FXS. Importantly, no studies have examined the trajectory of negative affect in females with FXS and, given that there are important sex differences in the prevalence and phenotype of FXS, this omission is a major gap in the literature. As such, it remains undetermined whether females with FXS, like their male peers, exhibit atypical negative affect early in life or whether their negative affect profiles more closely resemble typical trajectories.

There is some evidence to suggest that parent-mediated interventions improve infants’ regulation of negative affect in at-risk populations [37], and other work shows that there are sex differences in how parents socialize their children’s emotional expressions [38]. In addition to the potential for sex differences in intervention effects, females with FXS have, on average, higher cognitive and social-communicative abilities than males, putting them at differential risk for anxiety [3, 16]. Hence, a deeper understanding of how biological sex and temperament impact development and risk for comorbid psychiatric disorders in FXS is critical to understanding the unique needs for young children in this population and informing prevention programs and targeted treatments. Thus, the present study aimed to (1) determine whether early developmental trajectories of negative affect differ between FXS and TD, (2) examine sex effects on negative affect within FXS, and (3) investigate the relationship between negative affect to anxiety and ASD symptomatology in males and females with FXS. This study is among the first to examine biological sex differences in the association between negative affect and anxiety or ASD across infancy and preschool in FXS compared to TD using prospective longitudinal data. This is also the first study to examine the relationship between negative affect and later ASD and anxiety symptomatology in both males and females with FXS.

## Materials and methods

### Participants

The present study comprises a convenience sample of 185 children, 116 with FXS (75.0% male) and 69 with typical development (79.7% male), drawn from a series of longitudinal studies on early development at the University of South Carolina and the University of North Carolina. Participants were recruited primarily from research and medical sites as well as social media sites specializing in FXS or community parenting sites for TD recruitment. Inclusion criteria for all participants were (a) gestational age at least 37 weeks, (b) English as the primary language spoken in the home, and (c) and no other known medical conditions. Additionally, participants enrolled in the TD group were required to have no family history of ASD or other related disorders (e.g., FXS, tuberous sclerosis). Full mutation FXS was confirmed in all FXS participants through review of a genetic report indicating at least than 200 repeats of the CGG sequence on the *FMR1* gene. Demographic characteristics of the sample can be found in Table 1.

**Table 1:** Demographic characteristics of sample by percent of sample

	FXS (*n* = 116)	TD (*n* = 69)
Male (%)	75.0	79.7
Ethnicity (%)		
Hispanic/Latino	2.6	1.4
Not Hispanic/Latino	76.7	60.0
Unknown	20.7	38.6
Race (%)		
White	72.4	89.9
Black	5.2	5.8
More than one race	15.5	2.9
Other	1.7	0
Unknown	5.2	1.4

Approximately 29.9% of the male FXS participants in the present study overlap with a previous study [14]. The previous study did not include females or a control group. The present study sample was divided into three subsets to address each of the primary research questions. Samples for each research question are fully described in Table 3. Groups were matched by age for each model according to best practices in developmental disabilities research and did not significantly differ in age (*α* = 0.50; [39]).

### Procedures

Parents provided written informed consent prior to enrollment in the study. All procedures were approved by the Institutional Review Boards at the University of South Carolina and the University of North Carolina. Participants across all longitudinal research studies were assessed in a similar manner using a standard research protocol. Assessments occurred either in a research lab setting or in the child’s home. Children were assessed on a number of developmental outcomes, including temperament and ASD/anxiety symptomatology, at approximately 6, 9, 12, 18, 24, 36, 48, and 60 months of age. Diagnoses of FXS were confirmed via genetic report provided by a parent or confirmed through genetic analyses through the study, and TD controls were required to have no known genetic or familial risk factors for ASD or FXS.

Parent-report measures of negative affect and anxiety were completed using paper and pencil format and were mailed to parents prior to each assessment. Age-appropriate versions of parent-report measures were used. Behavioral measures of ASD symptoms and developmental ability were conducted by trained lab staff who were not blind to risk group. Measures of developmental ability and negative affect were collected at each time point, and parent-report measures of anxiety and ASD symptoms were collected at the 36, 48, or 60 month assessment.

### Measures

#### Negative affect

Temperamental negative affect was assessed using the negative affect composite score from the Rothbart Scales of Temperament. Parents completed the Infant Behavior Questionnaire-Revised (IBQ-R) for infants between 6 and 18 months [15], the Early Childhood Behavior Questionnaire (ECBQ) for children 18–36 months [40], and the Children’s Behavior Questionnaire (CBQ) for children 36–60 months [21]. These three measures retain a similar three-factor structure that comprises the three broad temperament domains, which demonstrate stability over time [40]. Importantly, this factor structure has been shown to be valid in populations with FXS [41]. The negative affect composite for all measures includes items related to anger, frustration, fear, sadness, soothability, and the ability to recover from distress. Mean negative affect score at each time point along with mean chronological age are presented in Table 2.

**Table 2:** Negative affect and participant characterization by age

	FXS	TD
6 months		
*n*	9	20
CA	6.16 (1.00)	6.18 (0.53)
IBQ-R NA score	2.64 (0.63)	2.91 (0.77)
9 months		
*n*	21	38
CA	9.26 (0.71)	9.16 (0.56)
IBQ-R NA score	2.92 (0.58)	3.18 (0.71)
12 months		
*n*	32	38
CA	12.28 (1.01)	12.29 (0.74)
IBQ-R NA score	3.12 (0.66)	3.13 (0.69)
18 months		
*n*	19	37
CA	17.65 (1.53)	18.08 (0.72)
ECBQ NA score	2.51 (0.58)	2.56 (0.41)
24 months		
*n*	32	30
CA	24.89 (1.39)	24.55 (1.11)
ECBQ NA score	2.56 (0.53)	2.54 (0.41)
36 months		
*n*	58	38
CA	36.76 (1.88)	36.57 (1.39)
CBQ NA score	3.58 (0.72)	3.37 (0.81)
48 months		
*n*	62	34
CA	47.05 (3.54)	46.49 (2.55)
CBQ NA score	3.83 (0.64)	3.77 (0.62)
60 months		
*n*	49	15
CA	57.50 (3.76)	57.05 (3.54)
CBQ NA score	3.81 (0.62)	3.49 (0.99)

*CA* chronological age, *IBQ-R* Infant Behavior Questionnaire-Revised, *NA* negative affect, *ECBQ* Early Childhood Behavior Questionnaire, *CBQ* Children’s Behavior Questionnaire

#### Generalized anxiety symptoms

Anxiety symptoms were assessed using the Spence Children’s Anxiety Scale for Parents (SCAS-P; [42]). The SCAS-P is a 38-item, parent-report questionnaire designed as a screening and diagnostic instrument for a number of anxiety disorders in young children. *T* scores for the generalized anxiety scale were utilized in the present study. *T* scores have a mean 50 and a standard deviation of 10. Higher scores represent more anxiety symptoms, and scores greater than one standard deviation from the mean represent elevated levels of anxiety. The generalized anxiety scale is a broad measure, and items that comprise this scale represent symptoms related to overall negativity (e.g., anger, sadness, and fear). Although this measure was not designed for use in populations with neurodevelopmental disabilities, it has been used in studies of children with ASD, intellectual disabilities, FXS, and other genetic syndromes [43, 44].

#### ASD symptoms

Severity of ASD symptomatology was measured using the Autism Diagnostic Observation Schedule, Second Edition (ADOS-2; [45]). The ADOS-2 is a play-based, semi-structured measure to elicit social interaction. Module 1, Module 2, Module 3, or the Toddler Module of the ADOS-2 was administered by lab-reliable or research-reliable examiners, and module selection was determined by the child’s age and expressive language abilities. Calibrated severity scores (CSS) were utilized in order to account for symptom severity across the modules that were used and to provide a validated, continuous measure of ASD symptomatology [46]. Scores range from 1 to 10, with higher values reflecting more severe ASD symptoms.

#### Developmental level

Developmental level was measured using the Mullen Scales of Early Learning (MSEL; [47]). The MSEL is a standardized assessment of early development for children from birth through 68 months. The MSEL comprises five domains: gross motor, visual reception, fine motor, receptive language, and expressive language. Correlational analyses were conducted to assess the relation between developmental ability (MSEL Early Learning Composite) and negative affect in each group (FXS and TD) at the first time point. Next, correlations between MSEL and negative affect at the first time point were run separately in males and females with FXS. Results from each of these correlations indicated that negative affect and developmental ability were not related (all *p*s > 0.05). Mullen ELC scores were thus used for descriptive purposes only and not included in the primary analyses.

### Analytic plan

Data were evaluated graphically using residual and Q-Q plots to evaluate the assumptions of linearity and normally distributed residuals. Levene’s test was used to assess the assumption of homogeneity of variance. Results from these analyses indicated that all assumptions were met. All analyses were run in R Statistical Computing Program (v3.3.1; [48, 49]). Descriptive statistics characterizing the sample for each research question can be found in Table 3. Age matching was conducted separately for each research question, so the samples vary slightly.

**Table 3:** Sample characterization by research question

Research Question #1				
	FXS		TD	
*n*	114		69	
Total number of observations	238		158	
Average observations per participant	2.10		2.29	
Mean age [months]	32.74 (17.74)		33.85 (13.86)	
Initial Mullen ELC	63.61 (16.83)		99.93 (11.71)	
Research Question #2				
	FXS		TD	
	Males	Females	Males	Females
*n*	87	29	55	14
Total number of observations	192	69	183	50
Average observations per participant	2.21	2.38	3.33	3.57
Mean age [months]	36.13 (17.18)	34.74 (16.81)	22.56 (14.49)	23.27 (13.66)
Initial Mullen ELC	58.21 (13.56)	78.55 (16.62)	99.75 (12.41)	100.64 (8.81)
Research Question #3				
	FXS Males		FXS Females	
*n*	23		11	
Predictors				
Total number of observations	92		50	
Average observations per participant	4.18		4.55	
Mean age [months]	28.80 (17.02)		27.44 (15.88)	
Initial Mullen ELC	68.17 (15.58)		86.73 (18.46)	
Outcome measures				
Mean age at outcome [months]	48.99 (12.6)		50.72 (6.37)	
SCAS-P GA total	44.76 (7.33)		49.55 (14.40)	
ADOS-2 CSS	5.57 (2.78)		4.56 (2.56)	

*ELC* Early Learning Composite, *ADOS-2* Autism Diagnostic Observation Schedule, Second Edition, *CSS* calibrated severity score, *SCAS-P* Spence Children’s Anxiety Scale for Parents, *GA* general anxiety

#### Research Question 1: Do children with FXS differ from TD children on developmental trajectories of negative affect?

We used Lme4 (Version #1.1–17) and lmerTest (Version #2.0–36; 51) to test group differences in negative affect across age using a multi-level model. Observations were nested within child and random effects were estimated for the intercept and slope. Group and age were entered in the model as level-2 predictors, with the TD group serving as the reference group. An age-by-group interaction was included to determine group differences in negative affect across age. Chronological age was centered at the grand mean of 32.86 months.

#### Research Question 2: Are there within group (FXS and TD) biological sex differences in developmental trajectories of negative affect?

We also used Lme4 (Version #1.1–17) to investigate the effects of sex on negative affect across age [50]. Due to power constraints related to sample size and model complexity, separate models were estimated for the FXS and TD groups to determine whether trajectories of negative affect differed by sex within each group. These models were estimated using random slopes and random intercept multi-level models with observations nested within child. Sex was included in the model as a level-2 predictor, and males were treated as the reference group. An age-by-sex interaction was also included in the model to determine age-related sex differences within each diagnostic group. Males and females were age-matched within groups, and chronological age was centered at the grand mean of participants’ ages for each group separately (*M*_FXS_ = 35.77 months; *M*_TD_ = 22.71 months).

#### Research Question 3: Within FXS, does negative affect predict anxiety or ASD symptomatology?

The third research question was addressed in two separate multiple regression models. In the first model, mean negative affect, sex, and a sex-by-negative affect interaction were entered as predictors, with SCAS-P scores entered as the outcome variable. In the second model, mean negative affect, sex, and a sex-by-negative affect interaction were entered as predictors and ASD ADOS-2 CSS scores were entered as the outcome variable. Participants for this analysis included a subset of children with FXS who had negative affect data from at least two time points and one outcome assessment of anxiety or autism symptoms between 48 and 60 months (*n* = 34, 67.6% male). For children who had multiple outcome measures in this time range (i.e., more than one SCAS-P or ADOS-2 score) the last available time point was used for analyses in order to take the most stable outcome measure into account. This sample did not differ from the overall FXS sample in either age, developmental ability, or negative affect.

## Results

### Research Question 1: Do children with FXS differ from TD children on developmental trajectories of negative affect?

**Fig. 1:**
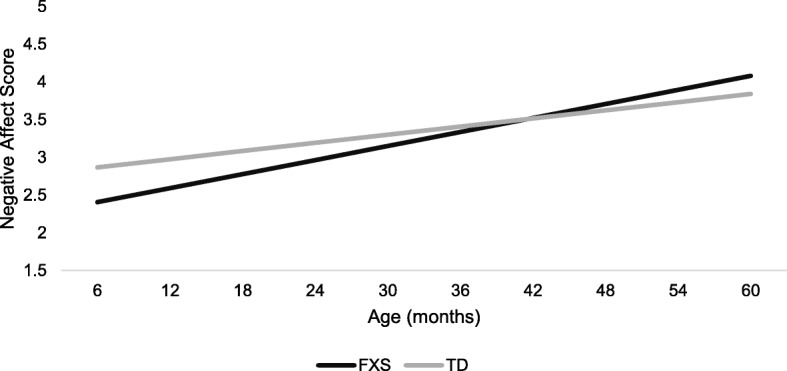
Model results for RQ1 illustrating group trajectories of negative affect over time

The first model tested the effect of the group on negative affect across age (see Table 4). Results indicated a significant effect of age (*b* = 0.018, *p* < .001) and a significant age-by-group interaction at 32.86 months, whereby for every 1-month increase in age, children with FXS experienced an increase in negative affect score that was 0.013 points higher relative to their TD peers (*b* = 0.013, *p* < .001). Results from probing the interaction indicated significant group differences emerged at 6 months (*b* = − 0.47, *p* = .003), but only remained until 36 months, (*b* = − 0.08, *p* = .386; see Fig. 1). This effect was such that at 6 months, children with FXS were predicted to score a half-point lower on negative affect than TD peers and this difference decreased across age. Thus, children with FXS appear to start off with lower negative affect than their TD peers but increase at a higher rate over time and by 36 months of age, at which point their negative affect becomes similar to that of their typically developing peers.

**Table 4:** Model results assessing the trajectory of negative affect in FXS and TD

	Estimate	SE	df	*t*	*p* value
Intercept	3.35	0.059	157.02	57.07	< .001
Age	0.018	0.0029	155.52	6.29	< .001
FXS	− 0.12	0.094	156.13	− 1.31	.191
Age × FXS	0.013	0.0046	124.10	2.74	.007

### Research Question #2: Are there within-group (FXS and TD) biological sex differences in developmental trajectories of negative affect?

**Fig. 2:**
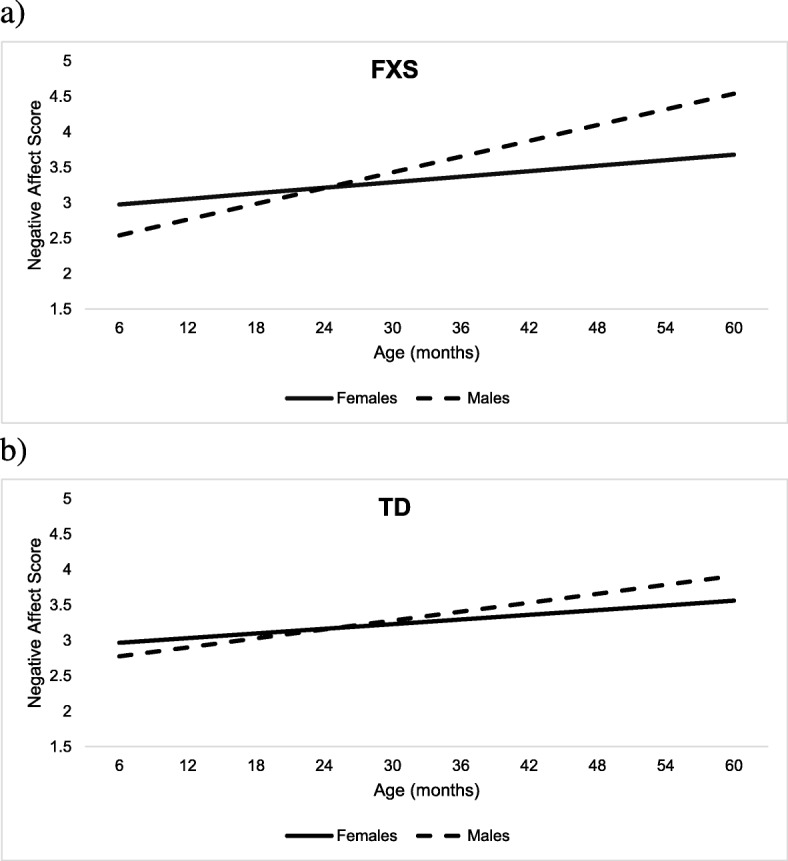
Model results for RQ2 illustrating trajectories of negative affect by sex in **a** FXS and **b** TD

The second research question was addressed by estimating separate models for the FXS and TD groups, given the known differential impact of sex on developmental trajectories in FXS. Each model tested differences in negative affect between males and females across age (see Tables 5 and 6). There was a significant difference between males and females with FXS (*b* = − 0.28, *p* = .024), such that females with FXS at 35.77 months were predicted to score approximately 0.30 points lower on negative affect than males with FXS of the same age. As evidenced by a significant age-by-sex interaction (*b* = − 0.024, *p* < .001), the predicted difference in the effect of age on negative affect was 0.024 points greater for males relative to females (see Fig. 2a). Results from probing the interaction indicated significant sex differences with females exhibiting more negative affect than males at 6 months (*b* = 0.43, *p* = .032); however, this sex difference was no longer significant by 12 months (*b* = 0.29, *p* = .101). Sex differences reemerged at 36 months (*b* = − 0.29, *p* = .021), with females being reported as having lower negative affect than males. In the TD model, there was only a significant effect of age (*b* = 0.02, *p* = .002) and not sex, such that for every 1-month increase in age, TD children exhibit a 0.02-point increase in negative affect (Fig. 2b). Thus, the developmental increase between 36 and 48 months (i.e., 12 months) is associated with a predicted 0.24-point increase in negative affect.

**Table 5:** Model results assessing the trajectory of negative affect by sex in children with FXS

	Estimate	SE	df	*t*	*p* value
Intercept	3.63	0.11	96.46	35.58	< .001
Age	0.037	0.0044	231.90	8.28	< .001
Female	− 0.28	0.12	96.08	− 2.30	.024
Age × female	− 0.024	0.0053	231.13	− 4.48	< .001

**Table 6:** Model results assessing the trajectory of negative affect by sex in TD children

	Estimate	SE	df	*t*	*p* value
Intercept	3.14	0.16	56.06	19.33	< .001
Age	0.021	0.0068	201.43	3.15	.002
Female	− 0.026	0.18	56.07	0.14	.886
Age × female	− 0.010	0.0077	208.71	− 1.31	.190

### Research Question #3: Within FXS, does negative affect predict anxiety or ASD symptomatology?

**Fig. 3:**
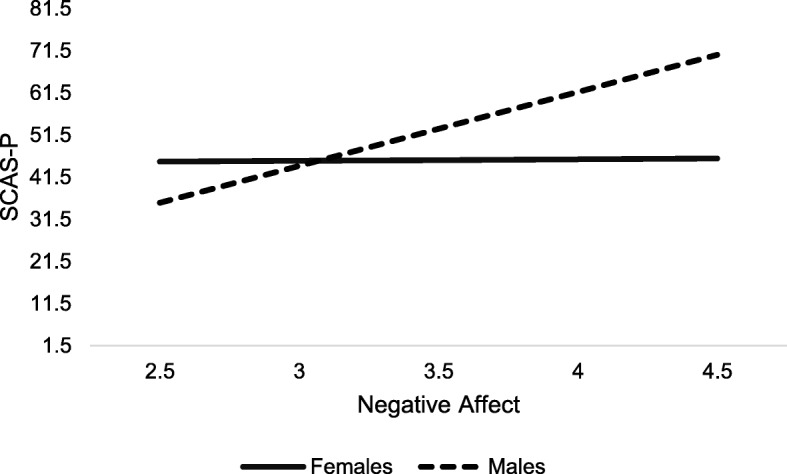
Model results for RQ3 illustrating sex by negative affect interaction in the prediction of SCAS-P scores

For the model predicting SCAS-P (anxiety) scores from early negative affect and sex, there were significant effects of negative affect (*b* = 17.55, *p* = .008) and sex (*b* = 52.70, *p* < .001), and a significant negative affect-by-sex interaction (*b* = − 17.18, *p* = .004). The interaction indicated a significant difference in effect between males and females, such that the rate of increase was 17 points greater for males relative to females (see Fig. 3). There were no significant effects for negative affect and sex on autism outcomes. Full model results are presented in Table 7.

**Table 7:** Model results assessing the prediction of anxiety and ASD symptoms by negative affect and sex in FXS

	SCAS-P				ADOS-2			
	Estimate	SE	*t*	*p* value	Estimate	SE	*t*	*p* value
Intercept	− 8.46	14.75	− 0.57	.57	− 1.65	4.48	− 0.37	.72
NA	17.55	4.40	2.86	.008	− 0.93	5.44	− 0.17	.87
Female	52.70	18.44	3.99	< .001	1.92	1.36	1.41	.17
NA × Female	− 17.18	5.52	− 3.11	.004	0.66	1.65	0.40	.69

*NA* negative affect

## Discussion

Negative affect is an early emerging temperament factor that encompasses fear, sadness, frustration, and difficulty regulating emotions [19]. Elevated and persistent negative affect predicts a range of poor outcomes including anxiety and ASD [24, 25]. Rates of anxiety and ASD are high and increasing in the general population [11, 51] and are acutely problematic in specific neurodevelopmental disorders. As such, efforts to identify early signs of anxiety and ASD have accelerated given the known benefits that early intervention can offer to reduce the occurrence or severity of these disorders. Recent work has begun to illuminate the complicated child and contextual factors that contribute to the emergence of developmental psychopathology such as anxiety and ASD. Because specific genetic populations like FXS are at elevated risk for specific co-morbid disorders and negative affect is presumed to be genetically influenced [18], studying FXS offers important insight into the potential child characteristics that contribute to developmental psychopathology. To this end, research work has increasingly focused on the characterization of anxiety and ASD in young children with FXS [4, 7, 52, 53]. The present study is one of only a few to evaluate sex differences in the negative affect trajectories in young children with FXS and their TD peers from 6 to 60 months of age. In addition, this project evaluates whether negative affect is differentially associated with anxiety and ASD symptoms between males and females with FXS. This study is an important next step in our understanding of emergent factors contributing to later psychopathology and sex-related differences in the phenotypic profile of FXS.

Findings suggested that both TD children and those with FXS exhibited increasing trajectories of negative affect from infancy through preschool. However, children with FXS showed steeper increases in negative affect with lower negative affect from 6 to 36 months of age and then levels commensurate with typical development from 36 months and older. This differential trajectory of increasing negative affect across age in the FXS group appears to be largely driven by sex effects, as males with FXS displayed significantly steeper trajectories than females. Specifically, male infants with FXS displayed lower negative affect than female infants with FXS at 6 months, equivalent levels at 12 months, and higher negative affect by 36 months. Age-related sex influences on negative affect trajectories were not observed in the TD group, a finding that corroborates prior work demonstrating that males and females in the general population do not differ in parent-reported negative affect [28].

The FXS-specific smaller increase in negative affect for females relative to males may reflect that females in general tend to exhibit milder presentations of FXS and related comorbidities across the board. This result highlights the importance of including both males and females in studies of early development and later psychopathology. This is especially critical in ascertaining a better understanding of early development in neurogenetic populations associated with intellectual disability, since we cannot infer that they follow patterns similar to the typically developing population. Including both males and females in FXS research can enhance our understanding of the unique vulnerabilities and needs of either sex.

The unique developmental trajectory of males with FXS characterized by lower negative affect in infancy followed by a shift to elevated levels across the early preschool years is noteworthy, as different patterns of association were found in TD males and females with FXS. This pattern is similar to earlier reports that sensory processing in male infants and toddlers with FXS shifts from “hypo-reactive” to “hyper-reactive” [54]. Likewise, heart activity has also been shown to shift from “under-aroused” to “hyper-aroused” in relation to elevated symptoms of ASD across a highly similar developmental window from infancy to early preschool [55]. These findings suggest a distinct developmental pattern of both behavioral and physiological trajectories that parallel brain development [56]. Although no physiological markers of early reactivity were measured in the present study, this finding fits in with theories suggesting that atypical outcomes in FXS are associated with a shift from under- to over-arousal from infancy through early childhood [55]. Furthermore, studies of trajectories of anxiety in the general population suggest that physiological characteristics contribute to anxiety risk differently in males and females [24]. Collectively, these studies highlight the importance of adopting a longitudinal approach as mean levels at a single age may mask critical patterns of stability or change that represent unique predictive power. Our findings illustrate that subtle differences in negative affect among children with FXS and those with TD vary across the first few years of life.

Our results also suggest that among children with FXS, negative affect predicts anxiety symptoms, but not ASD symptoms. These findings are consistent with previous work that examined the longitudinal relation between parent-reported negative affect and anxiety and ASD in males with FXS only [14]. Importantly, however, the current study found that sex was differentially related to the effect of negative affect on later anxiety symptoms. In males with FXS, there appears to be a positive relationship between negative affect and anxiety, whereas this effect seems to be weaker in females (Fig. 3). Females with FXS may exhibit different risk factors for later anxiety than their male peers, such as less impaired social-communicative and/or cognitive abilities [3, 16]. It is possible that females’ cognitive and social abilities relative to males may enable them to better learn and utilize effective coping skills that are important to the prevention of childhood anxiety [57]. It is of continued importance to characterize the phenotypic variability in females with FXS and evaluate how this variability may differentially affect later adjustment or disorders.

Despite work that has highlighted negative affect as a predictor of ASD in infants with familial risk for ASD (e.g., an older sibling with ASD), this relation does not seem to hold in children at elevated risk for ASD due to FXS. Although there is a large body of work suggesting emotion regulation difficulties in ASD may relate to the emergence of comorbid anxiety [58], no study to date has specifically examined whether negative affect predicts anxiety in a population of children with non-syndromic ASD. Thus, the question of whether there is a common etiological pathway between negative affect and anxiety within FXS and non-syndromic ASD warrants further exploration. The current findings also contribute to the debate regarding the independence and overlap of symptoms of anxiety to ASD in FXS as elevated anxiety in FXS is often attributed as the cause of “misdiagnoses of ASD” [59]. Social avoidance and impaired eye contact are hallmark features of both anxiety and ASD in FXS, and these overlapping features are often misattributed as ASD symptoms without taking into account the contributions of anxiety [59, 60]. Our findings that trajectories of negative affect predicted anxiety and not ASD suggests that anxiety and ASD are indeed independent disorders with different risk factors. Further, these findings support the predictive power of trajectories relative to a single time point when studying comorbidities in neurodevelopmental disorders [14, 25].

Future work should also investigate how the regulatory components of temperament, such as effortful control, may moderate not only the expression of negative emotion in FXS, but also the relation between early negative affect and later outcomes. There are numerous etiological and phenotypic differences between non-syndromic ASD and FXS with comorbid ASD, and it is unlikely that these differences are solely related to intellectual disability in FXS. We found no relation between negative affect and developmental level, and other studies have shown that there is no difference in components of negative affect between clinic-referred toddlers with ASD and developmental delays and those with non-syndromic developmental delay [33]. Thus, it is possible that young children with FXS have a genetic predisposition towards developing additional comorbid conditions over and above the contributions of intellectual disability.

The present study furthers our understanding of the emergence of psychological disorders in at-risk populations, but it is not without some limitations. First, only parent-report measures of negative affect were utilized. Although parent-report instruments offer important information about how young children interact with their environment on a day-to-day basis, they are blunt measures that may not be sensitive enough to capture low-level differences in temperamental regulation which may have potential to distinguish the groups included in this study. In addition, due to the low incidence of FXS, the current study was underpowered to simultaneously test the multiplicative effects of age group and sex longitudinally between both FXS and TD groups. Finally, largely due to the rarity of FXS, the sample that has been clinically characterized for Research Question #3 is quite small. As a result, our current sample may not be representative of the entire population of children with FXS. Further, our data do not allow us to predict change over time in anxiety or ASD symptomatology. This offers an important avenue for future work. We also note that the SCAS-P was designed and normed to measure anxiety in neurotypical children, although it has been used in neurodevelopmental disorders.

Nevertheless, our findings offer a number of important future directions. Research should continue to explore alternative pathways to outcomes for psychopathology, such as attention or physiological regulation, to increase our understanding of early signs and underlying mechanisms associated with ASD and anxiety in FXS.

## Conclusions

The present study examined the trajectory of negative affect from infancy through preschool in males and females with FXS and TD and its relation to anxiety and ASD. Results revealed a complex relationship between group, development, and sex effects. The current work illustrates the unique mechanistic questions that can be addressed by studying the development of psychopathology in a specific neurogenetic population. Importantly, the present study indicates that, as in neurotypical populations, temperamental negative affect can be an important early marker for anxiety in young children with FXS. However, females with FXS as well as a subset of males with high negative affect may be especially susceptible to comorbid anxiety symptoms. Given the clear effectiveness of early intervention on later anxiety symptoms efforts [61], these results provide opportunities for the pursuit of an important target for early detection and intervention.

## Data Availability

The datasets analyzed during the current study are available from the corresponding author on reasonable request.

## Abbreviations

ADOS-2: Autism Diagnostic Observation Schedule, Second Edition
ASD: Autism spectrum disorder
CBQ: Children’s Behavior Questionnaire
CSS: Calibrated severity score
ECBQ: Early Childhood Behavior Questionnaire
ELC: Early Learning Composite
FMRP: Fragile X mental retardation protein
FXS: Fragile X syndrome
IBQ-R: Infant Behavior Questionnaire-Revised
MSEL: Mullen Scales of Early Learning
SCAS-P: Spence Children’s Anxiety Scale for Parents
TD: Typical development
